# Multi-Target Anticancer Activity of Structurally Diverse Schiff Bases: Insights into Cell-Cycle Arrest, DNA Damage, Metabolic Signaling, and Biomolecular Binding

**DOI:** 10.3390/cimb48010057

**Published:** 2026-01-01

**Authors:** Nenad Joksimović, Jelena Petronijević, Ignjat Filipović, Nenad Janković, Bojana Ilić, Tatjana Stanojković, Ana Djurić

**Affiliations:** 1 Department of Chemistry, Faculty of Science, University of Kragujevac, Radoja Domanovića 12, 34000 Kragujevac, Serbia; jelena.petronijevic@pmf.kg.ac.rs (J.P.); ignjat.filipovic@pmf.kg.ac.rs (I.F.); 2 Department of Sciences, Institute for Information Technologies Kragujevac, University of Kragujevac, Jovana Cvijića bb, 34000 Kragujevac, Serbia; nenad.jankovic@uni.kg.ac.rs; 3 Clinic for Endocrinology, Diabetes and Metabolic Diseases, University Clinical Centre of Serbia, 11000 Belgrade, Serbia; ilicbbojana@gmail.com (B.I.); ana.jelicmariokov@gmail.com (A.D.); 4 Institute of Oncology and Radiology of Serbia, Pasterova 14, 11000 Belgrade, Serbia; stanojkovict@ncrc.ac.rs

**Keywords:** Schiff bases, anticancer activity, cell cycle arrest, DNA damage, ROS modulation, HIF-1α, PDK3, bovine serum albumin, molecular docking

## Abstract

Schiff bases are widely studied for their biological activities, yet structure–activity relationships governing their anticancer potential remain insufficiently understood. In this work, eight structurally diverse imine derivatives (**A**–**H**) were evaluated for their cytotoxic, biochemical, and biomolecular interactions in human cancer cells. Their antiproliferative effects were assessed in HeLa, A549, and LS174T cell lines, with MRC-5 fibroblasts used as a non-malignant control. Cytotoxicity screening identified three compounds (**A**, **B**, and **F**) with the highest potency, prompting further mechanistic investigation. Cell cycle analysis revealed G1 arrest and accumulation of sub-G1 populations for all three derivatives, with compound **B** additionally increasing S-phase content and compound **F** inducing G2/M arrest. All compounds reduced intracellular ROS levels and caused significant DNA damage at subtoxic concentrations. Western blot analysis demonstrated downregulation of HIF-1α and PDK3, suggesting disruption of hypoxia-associated metabolic signaling. Fluorescence quenching experiments showed strong binding of the active compounds to bovine serum albumin (K_a_ ≈ 10^6^ M^−1^), and molecular docking supported stable interactions near tryptophan-adjacent binding regions. Collectively, these findings indicate that selected Schiff bases exert multi-target anticancer activity by modulating oxidative stress, DNA integrity, cell-cycle progression, and metabolic adaptation pathways, warranting further investigation of their therapeutic potential.

## 1. Introduction

In recent decades, the global incidence of various life-threatening diseases has increased significantly, posing a serious threat to human health and mortality. Advances in biomedical research have enabled the discovery and development of numerous therapeutic agents for the treatment of many severe disorders. However, for several complex diseases, including cancer, which remains one of the leading causes of death worldwide, the development of effective and selective therapeutic strategies is still a major medical challenge. Consequently, extensive research efforts have been directed toward discovering novel anticancer agents with improved efficacy and safety profiles [1,2,3,4,5]. The discovery of cisplatin marked a revolutionary advancement in chemotherapy and significantly improved the treatment outcomes of various malignancies [6]. Nevertheless, its clinical application is restricted by the development of drug resistance and numerous dose-limiting side effects [7,8,9,10,11]. Therefore, the design and synthesis of new anticancer agents with mechanisms of action similar to cisplatin but reduced toxicity represent a current priority in medicinal chemistry.

In this context, significant research has focused on metal–organic complexes as well as organic molecules as potential cisplatin alternatives [12,13,14,15]. Among them, imines, also known as Schiff bases, have emerged as promising candidates due to their structural diversity and broad spectrum of biological activities [16]. Amines and their derivatives are widely applied in various industrial fields, including as polymer stabilizers, dye intermediates, and catalysts in organic synthesis [17]. Furthermore, numerous Schiff base derivatives have demonstrated notable antitumor, antibacterial, antifungal, antioxidant, anti-inflammatory, antiviral, antiproliferative, and antipyretic properties [16,18].

Previous studies have emphasized the key role of amino groups in modulating the biological activity of Schiff base derivatives, highlighting their importance in biomolecular interactions and pharmacological potential [19,20,21]. In addition to their biological relevance, these compounds are attractive synthetic targets because of their straightforward preparation, which does not typically require harsh reaction conditions. Imines are usually obtained by reaction between aldehydes or ketones and amines in a polar solvent [22]. Since the biological activity of a compound is influenced not only by its cytotoxic effects but also by its ability to interact with biomacromolecules, it is important to investigated their binding affinity towards serum transport proteins. Bovine serum albumin (BSA) is generally selected as a model protein due to its structural similarity to human serum albumin (HSA) [23,24].

In this study, we evaluated eight structurally diverse Schiff bases for their anticancer potential. We focused on cellular mechanisms including cytotoxicity, cell-cycle arrest, ROS modulation, DNA damage, and metabolic pathway regulation, complemented by protein interaction studies with BSA and molecular docking analyses. Our integrated approach aimed to identify promising multi-target compounds with favorable pharmacological profiles and mechanistic insights relevant to anticancer drug development.

## 2. Materials and Methods

### 2.1. General

All chemicals, including solvents and starting materials, were purchased from Sigma-Aldrich (St. Louis, MO, USA) and used without further purification unless otherwise stated. Melting points of the synthesized compounds were measured using a Mel-Temp apparatus (Electrothermal, Staffordshire, UK) and are reported as uncorrected values. Infrared (IR) spectra were recorded on a Perkin–Elmer Spectrum One FT-IR spectrophotometer (PerkinElmer, Llantrisant, UK) using KBr pellets. Nuclear magnetic resonance (NMR) spectra were acquired in DMSO-d_6_ with tetramethylsilane (TMS) as an internal reference on a Varian Gemini 200 MHz spectrometer (Varian Associates Inc., Palo Alto, CA, USA). Elemental analyses (C, H, N) were carried out using a Carlo Erba EA 1108 analyzer (Carlo Erba Strumentazione, Milan, Italy). Fluorescence measurements were performed using a Shimadzu RF-1501 PC spectrofluorometer (Shimadzu Corporation, Kyoto, Japan).

### 2.2. Cell Lines

In this in vitro study, the cytotoxic effects of the tested extracts were assessed using four cell lines: normal human embryonic lung fibroblasts (MRC-5) and three human cancer cell lines—cervical adenocarcinoma (HeLa), lung adenocarcinoma (A549), and colon adenocarcinoma (LS174T). The cells were cultured in RPMI-1640 medium supplemented with 3 mM L-glutamine, 100 μg/mL streptomycin, 100 IU/mL penicillin, 10% heat-inactivated fetal bovine serum (56 °C), and 25 mM HEPES buffer. The pH of the medium was adjusted to 7.2 using a bicarbonate solution. All cell lines were maintained at 37 °C in a humidified incubator under a 5% CO_2_ atmosphere. The cancer cell lines were obtained from the American Type Culture Collection (ATCC, Manassas, VA, USA), while RPMI-1640 medium, L-glutamine, and HEPES were purchased from PAA Laboratories (Pasching, Austria).

### 2.3. MTT Assay for Cell Viability

The cytotoxic effects of the investigated extracts on cell viability were assessed using the MTT assay (3-(4,5-dimethylthiazol-2-yl)-2,5-diphenyltetrazolium bromide), originally described by Mosmann [25] and subsequently modified by Ohno and Abe [26]. This colorimetric method is based on the reduction of the yellow tetrazolium salt (MTT) to insoluble purple formazan crystals by metabolically active cells. The quantity of formazan produced, which is proportional to the activity of intracellular oxidoreductase enzymes such as mitochondrial dehydrogenases, directly reflects the number of viable cells.

HeLa, MRC-5, A549, and LS174T cells were seeded in 96-well plates at initial densities of 3000, 5000, 5000, and 7000 cells per well, respectively. After a 24 h incubation period to permit cell attachment, the cultures were exposed to five serial concentrations of the test extracts (12.5–400 μg/mL). Following 72 h of treatment, 20 μL of MTT solution (5 mg/mL in phosphate-buffered saline, PBS) was added to each well, and plates were incubated for an additional 4 h at 37 °C in a 5% CO_2_ humidified atmosphere. Formazan crystals formed during incubation were solubilized by adding 100 μL of 10% sodium dodecyl sulfate (SDS), after which absorbance was recorded at 570 nm following a 24 h dissolution period.

Cell viability (%) was calculated relative to untreated control cells according to the following equation: (A_treated_/A_control_) × 100. The half-maximal inhibitory concentration (IC_50_) was defined as the concentration of extract that reduced cell viability by 50% compared to control conditions. All experiments were carried out in triplicate to ensure reproducibility of results.

### 2.4. Cell Cycle Analysis by Flow Cytometry

Human cervical carcinoma HeLa cells were exposed to compounds **A**, **B**, and **F** for 24 h and 48 h at concentrations equivalent to their respective IC_50_ and 2IC_50_ values, which were previously determined following 72 h of treatment. After incubation, cells were harvested, rinsed with phosphate-buffered saline (PBS), and fixed in 70% ethanol as described in the established protocol [27]. Fixed samples were stored at −20 °C for a minimum of one week prior to further analysis. For cell cycle assessment, ethanol-fixed cells were centrifuged, washed, and resuspended in PBS supplemented with RNase A, followed by incubation for 30 min at 37 °C to remove residual RNA. Cells were subsequently stained with propidium iodide. The distribution of cells across different phases of the cell cycle was quantified using a BD FACSCalibur flow cytometer (BD Biosciences, San Jose, CA, USA). Data from 10,000 gated events per sample were acquired and analyzed using the CELLQuest 3.3 software.

### 2.5. Evaluation of ROS Production

Intracellular reactive oxygen species (ROS) generation in HeLa cells was assessed after 24 h of treatment with subcytotoxic concentrations (IC_20_) of compounds **A**, **B**, and **F**, as previously established using the MTT assay. Following treatment, cells were harvested, rinsed with phosphate-buffered saline (PBS), and incubated with 2′,7′-dichlorodihydrofluorescein diacetate (H_2_DCFDA, 30 μM in PBS; Sigma-Aldrich) for 45 min at 37 °C in accordance with a previously described protocol [28]. After staining, excess probe was removed by washing with PBS. The fluorescence intensity of the oxidized product, dichlorofluorescein, which reflects ROS levels, was quantified using a BD FACSCalibur flow cytometer (BD Biosciences, Franklin Lakes, NJ, USA). For each sample, 20,000 cellular events were collected and analyzed using CELLQuest software (BD Biosciences).

### 2.6. Evaluation of DNA Damage by Comet Assay

HeLa human cervical carcinoma cells were exposed to IC_20_ concentrations of compounds **A**, **B**, and **F** for 24 h. Following treatment, cells were harvested, washed with phosphate-buffered saline (PBS), resuspended in a cryoprotective medium (RPMI supplemented with 10% dimethyl sulfoxide (DMSO) and 20% fetal calf serum (FCS)), and stored at −80 °C until analysis. DNA strand breaks were quantified using the alkaline single-cell gel electrophoresis (comet) assay, performed according to a previously established protocol [29].

For analysis, frozen samples were rapidly thawed by adding 1 mL of PBS to 0.5 mL aliquots, followed by centrifugation at 2000 rpm for 10 min at 4 °C. The resulting cell pellets were washed twice with PBS and resuspended to a final density of 2.5 × 10^5^ cells/mL. An aliquot of 30 µL of the cell suspension was gently mixed with 140 µL of 1% low-melting-point (LMP) agarose (37 °C), and twelve 10 µL drops of the mixture were dispensed onto slides precoated with normal-melting-point (NMP) agarose using a 12-gel chamber template. The embedded cells were lysed for 1 h at 4 °C in lysis buffer (2.5 M NaCl, 0.1 M Na_2_EDTA, 10 mM Tris, 1% Triton X-100, pH 10). Slides were subsequently incubated in alkaline electrophoresis buffer (0.3 M NaOH, 0.001 M Na_2_EDTA) for 20 min to allow DNA unwinding, followed by electrophoresis at 1 V/cm for 30 min at 4 °C.

After electrophoresis, slides were neutralized in PBS for 10 min at 4 °C, fixed sequentially in 70% ethanol and absolute ethanol (10 min each), and air-dried. DNA was visualized by staining with SYB^TM^ Gold (Invitrogen, Carlsbad, CA, USA) for 30 min in the dark, followed by two brief rinses in distilled water. Comet images were captured and analyzed using Comet Assay IV software (Perceptive Instruments, version 4.3.0), and DNA damage was expressed as tail intensity (% DNA in tail) based on measurements of 50 nucleoids per gel.

### 2.7. Evaluation of HIF-1α and PDK-3 Expression by Western Blot

HeLa human cervical carcinoma cells were seeded into six-well plates at a density of 0.5 × 10^6^ cells per well in 2 mL of complete culture medium and allowed to adhere for 24 h at 37 °C. Cells were then treated for 24 h with compounds at concentrations corresponding to their IC_50_ and IC_20_ values. Following treatment, cells were rinsed three times with ice-cold phosphate-buffered saline (PBS), detached by scraping, and pelleted by centrifugation at 3000 rpm for 5 min. The resulting pellets were lysed in RIPA buffer supplemented with cOmplet^TM^ EDTA-free Protease Inhibitor Cocktail (Roche Applied Science, Mannheim, Germany) and incubated on ice for 1 h. Cell disruption was further enhanced by sonication (three 10 s cycles with 30 s cooling intervals). Lysates were clarified by centrifugation at 11,000 rpm for 20 min at 4 °C, and the supernatants were transferred to fresh tubes. Total protein concentrations were quantified using a BCA Protein Assay Kit (Thermo Fisher Scientific, Waltham, MA, USA).

Equal amounts of protein were mixed with loading buffer, denatured at 90 °C for 10 min, separated by SDS–PAGE using 10% Tris–glycine gels, and electrotransferred onto nitrocellulose membranes. Membranes were blocked with 5% non-fat dry milk prepared in TBST (Tris-buffered saline with 0.1% Tween 20) for 1 h at room temperature, followed by overnight incubation at 4 °C with primary antibodies: anti-PDK3 (1:1000, ab154549, Abcam, Cambridge, UK), anti-HIF-1α (1:300, Santa Cruz Biotechnology, Heidelberg, Germany), and anti-β-actin (1:1000, ab3280, Abcam, Cambridge, UK). After three washes with TBST, membranes were incubated for 1 h with horseradish peroxidase (HRP)-conjugated secondary antibodies (Lumi-LightPLUS Western Blotting Kit, Roche Applied Science, Penzberg, Germany). Following additional washing, protein bands were detected by chemiluminescence using the ChemiDoc Imaging System (Bio-Rad, Hercules, CA, USA). Band intensities were quantified with Image Lab 6.0.1 software (Bio-Rad), and protein expression levels were normalized against β-actin.

### 2.8. BSA Fluorescence Binding Study

A 100 μM solution of bovine serum albumin (BSA) was prepared in 10 mM phosphate-buffered saline (PBS, pH 7.4) and stored in the dark at 5 °C for no longer than 12 h. Complexes of BSA with compounds **A**, **B**, or **F** were prepared individually by mixing a fixed concentration of BSA with increasing concentrations of each compound. The molar ratios of BSA to compounds were set as 1:0 (control), 1:0.5, 1:1, 1:1.5, 1:2, 1:2.5, and 1:3, in a total volume of 5.0 mL at pH 7.4. Samples were incubated at 25 °C for 6 h to allow complex formation. The final concentration of BSA in each sample was 10 μM, while the concentrations of compounds **A**, **B**, and **F** ranged from 0.5 μM to 30 μM. Fluorescence emission spectra were recorded immediately after incubation upon excitation at 280 nm, and emission intensity was measured over the 300–500 nm range.

### 2.9. Molecular Docking

Structures of investigated compounds were generated using DS Visualizer v20.1.0.19295, their structures optimized using MOPAC2016 19.038W [30] and prepared for docking experiments using AutoDockTools 1.5.6. Docking experiments were performed using AutoDock 4.2.6 [31]. Target molecule was obtained from crystal structure designated 4or0 on rscb.org site [32]. Co-crystalized water was removed. Co-crystalized substrate (NPX) was removed and its structure, along with optimized structures of **A**, **B**, and **F** were prepared for experiments by marking rotatable bonds, calculating Gasteiger charges and merging non-polar hydrogen atoms. Sites were defined using cubes with edges of 60 points (0.375 Angstrom per point), centered on residues TRP134 and TRP213 of the target molecule. Docking of each compound included 10 genetic algorithm runs with 2.5 × 10^7^ energy evaluations, 150 individuals in population, 27,000 max generations and rest of the parameters left at default values.

## 3. Results

### 3.1. Anticancer Evaluation

**Figure 1:**
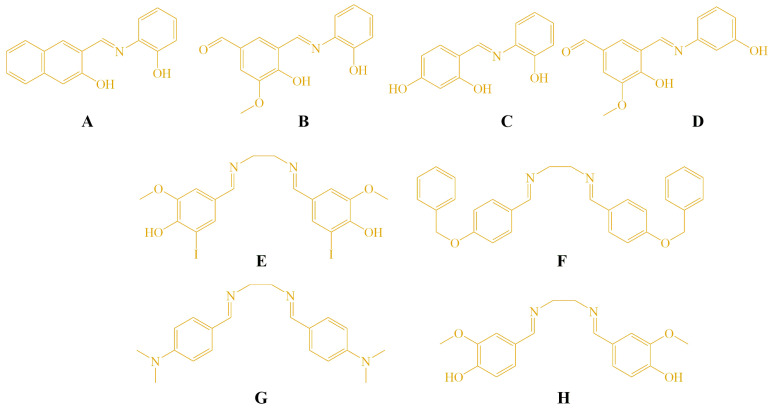
Structures of the tested compounds **A**–**H.**

The molecular structures of the Schiff bases (**A**–**H**) evaluated in this study are shown in Figure 1. These compounds were selected based on their structural diversity and the known biological relevance of imine-containing molecules. The tested compounds **C**–**H** were synthesized previously, and the method for synthesis is described in our earlier publication [22]. Additionally, two derivatives (**A** and **B**) were synthesized using the same procedure. The aldehyde and amine were refluxed in ethanol, and the formation of the Schiff base proceeded smoothly without the addition of glacial acetic acid or any other catalyst. Compound **A** has been previously reported in the literature [33], while compound **B** represents a newly synthesized derivative prepared using the same synthetic protocol. The reaction scheme and spectroscopic characterization of compounds **A** and **B** are provided in the ESI (Scheme S1, Figures S1–S5).

Their in vitro anticancer activity was assessed against three human cancer cell lines representing distinct tissue origins—cervical adenocarcinoma (HeLa), lung adenocarcinoma (A549), and colon adenocarcinoma (LS174T)—as well as against the non-malignant human embryonic lung fibroblast cell line (MRC-5), used as a model of normal cells.

HeLa, A549, and LS174T cell lines were selected as representative models of cervical, lung, and colorectal cancers, respectively. This selection allows preliminary evaluation of anticancer potential across different tissue types and provides a foundation for mechanistic insights. Studies on additional cancer cell lines are planned to further investigate tissue-specific responses and potential mechanisms of action.

### 3.2. Cytotoxic Activity

The cytotoxic activity of the compounds was evaluated after 72 h of incubation in three malignant cell lines (HeLa, LS174T, A549) and the non-malignant MRC-5 cell line. The derivatives exhibited mild to moderate cytotoxicity toward the cancer cells, with IC_50_ values ranging from 39.45 ± 0.20 μM to 130.68 ± 6.79 μM, with HeLa cells showing the highest sensitivity. Compounds **A**, **B**, and **F** demonstrated the most prominent antiproliferative effects (Table 1).

Because of their potency, compounds **A**, **B**, and **F** were additionally evaluated after 24 h of exposure in HeLa cells (Table 2). All three compounds showed lower cytotoxicity toward MRC-5 cells than toward the malignant cell lines, suggesting a favorable selectivity profile. The observed cytotoxicity agrees with earlier reports indicating that molecules containing hydroxyphenyl or benzyloxy fragments exhibit notable anticancer activity [2,14,15].

### 3.3. Cell Cycle Analysis

**Figure 2:**
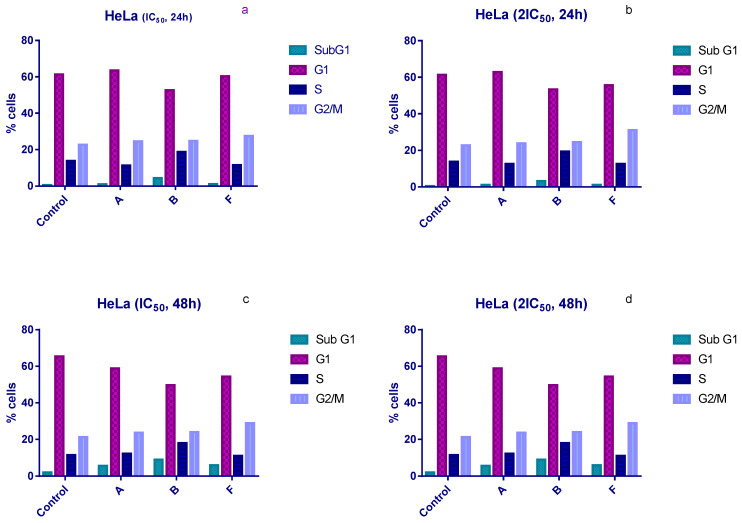
Changes in the cell cycle of HeLa cells after treatment with tested compounds. (**a**) HeLa cells treated with IC_50_ concentrations for 24 h; (**b**) HeLa cells treated with 2×IC_50_ concentrations for 24 h; (**c**) HeLa cells treated with IC_50_ concentrations for 48 h; (**d**) HeLa cells treated with 2×IC_50_ concentrations for 48 h.

Cell cycle distribution was examined following 24 h and 48 h of treatment with IC_50_ and 2 × IC_50_ concentrations of compounds **A**, **B**, and **F**. All three compounds induced an accumulation of HeLa cells in the sub-G1 phase and a corresponding arrest in the G1 phase compared with untreated controls (Figure 2).

Compound **B** produced the most pronounced effects. After 24 h of exposure, the proportion of sub-G1 cells increased to 4.5% and 3.25% at IC_50_ and 2 × IC_50_, respectively, compared with 0.5% in the control (Figure 2a,b). After 48 h, sub-G1 levels rose further (7.8% and 9% at IC_50_ and 2 × IC_50_), compared with 2% in untreated cells (Figure 2c,d). Compound **B** also increased the proportion of cells in S phase under all experimental conditions, whereas compounds **A** and **F** did not induce significant S-phase changes.

In contrast, compound **F** induced an accumulation of HeLa cells in the G2/M phase after 24 h. Treatment with IC_50_ and 2 × IC_50_ concentrations increased the G2/M population to 27.5% and 31%, respectively, compared to 22.8% in controls (Figure 2a,b).

### 3.4. Analyzes of ROS Level

**Figure 3:**
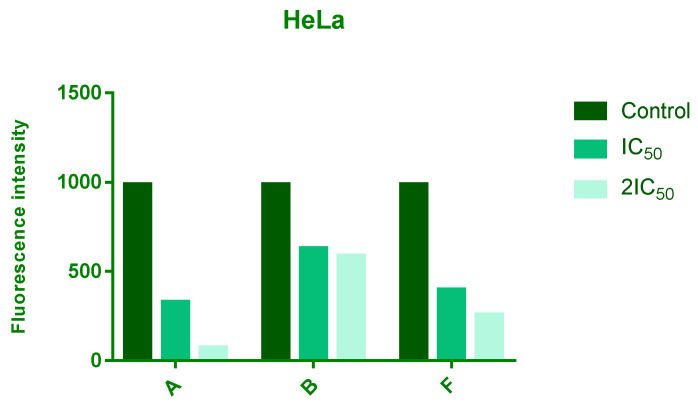
Effects of 24 h treatment of HeLa cells with IC_50_ and 2IC_50_ concentrations of tested compounds on endogenous ROS levels. The intensity of fluorescence corresponds to level of a ROS.

ROS levels were assessed after 24 h of treatment with IC_50_ and 2 × IC_50_ concentrations of compounds **A**, **B**, and **F**. All three compounds reduced intracellular ROS levels in a dose-dependent manner, with compound **A** showing the most pronounced effect, followed by **F** and **B** (Figure 3).

### 3.5. DNA Damage Analysis

**Figure 4:**
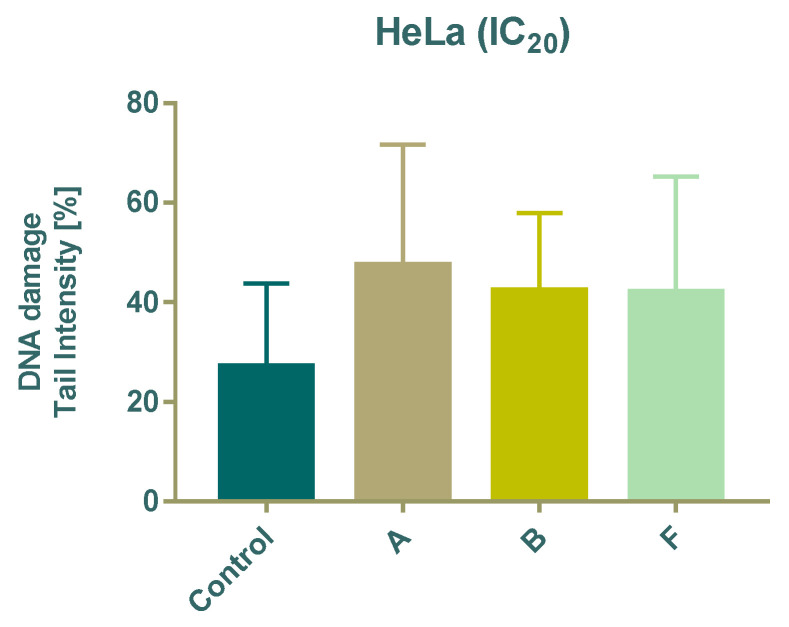
Effect of 24 h treatment of tested compounds on DNA damage in HeLa cells using the Comet assay.

DNA damage was evaluated using the comet assay after 24 h treatment with subtoxic (IC_20_) concentrations of compounds **A**, **B**, and **F**. All tested compounds significantly increased DNA damage compared with untreated cells, as indicated by a higher percentage of DNA in the comet tail (Figure 4).

### 3.6. Western Blot Analyses

**Figure 5:**
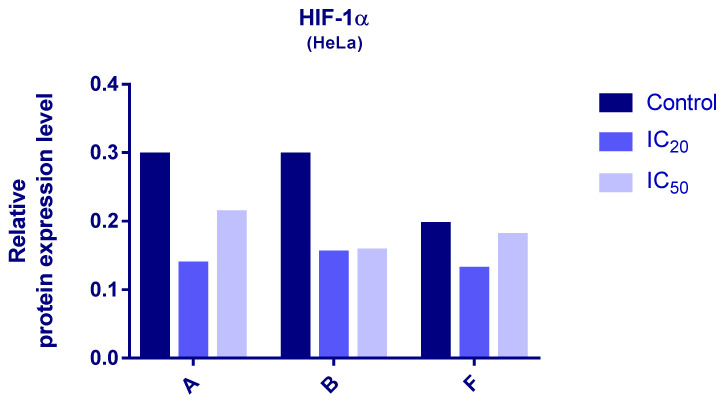
Expression of HIF-1α in HeLa cells after 24 h treatment with tested compounds (concentrations correspond to IC_20_ and IC_50_).

**Figure 6:**
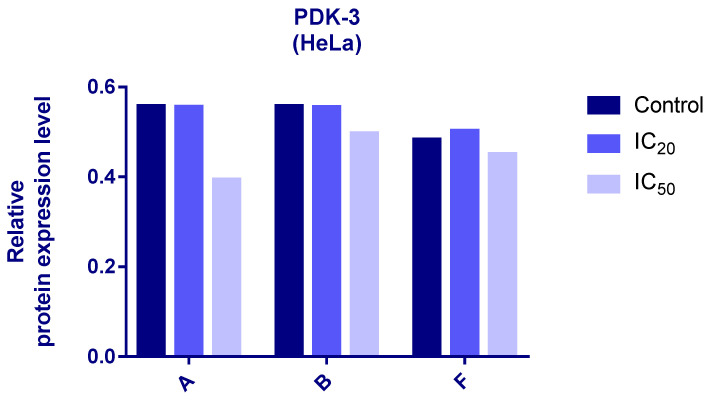
Expression of PDK-3 in HeLa cells after 24 h treatment with tested compounds (concentrations correspond to IC_20_ and IC_50_).

**Figure 7:**
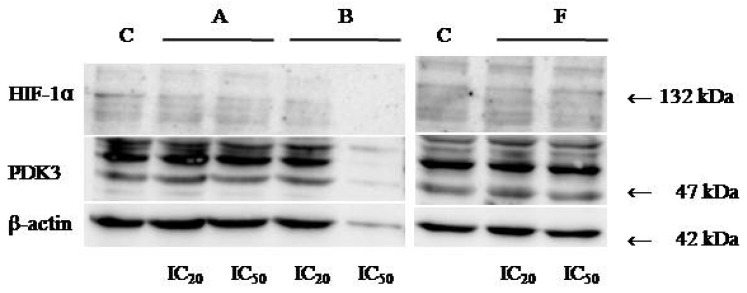
Immunoblots for HIF-1α, PDK-3, and β-actin of HeLa whole-cell lysates after 24 h treatment with tested compounds (concentrations correspond to IC_20_ and IC_50_).

Protein expression levels of HIF-1α and PDK3 (normalized to β-actin) were quantified in HeLa cells following 24 h treatment with compounds **A**, **B**, and **F** at IC_20_ and IC_50_ concentrations. Representative immunoblots and quantifications are provided in Figure 5, Figure 6 and Figure 7.

Cells treated with compounds **A** and **B** exhibited reduced expression of both HIF-1α and PDK3 at IC_20_ and IC_50_ compared to controls. HIF-1α levels increased slightly at IC_50_ relative to IC_20_, while PDK3 demonstrated the opposite trend. Notably, β-actin levels were markedly reduced in cells treated with compound **B**, which may reflect high cytotoxicity. Therefore, the normalized expression data for compound **B** should be interpreted with caution.

In cells treated with compound **F**, PDK3 expression was higher at IC_20_ compared to IC_50_ and controls. Compound **F** also reduced HIF-1α expression, especially at IC_20_

### 3.7. Interactions of Compounds **A**, **B**, and **F** with Bovine Serum Albumin

**Figure 8:**
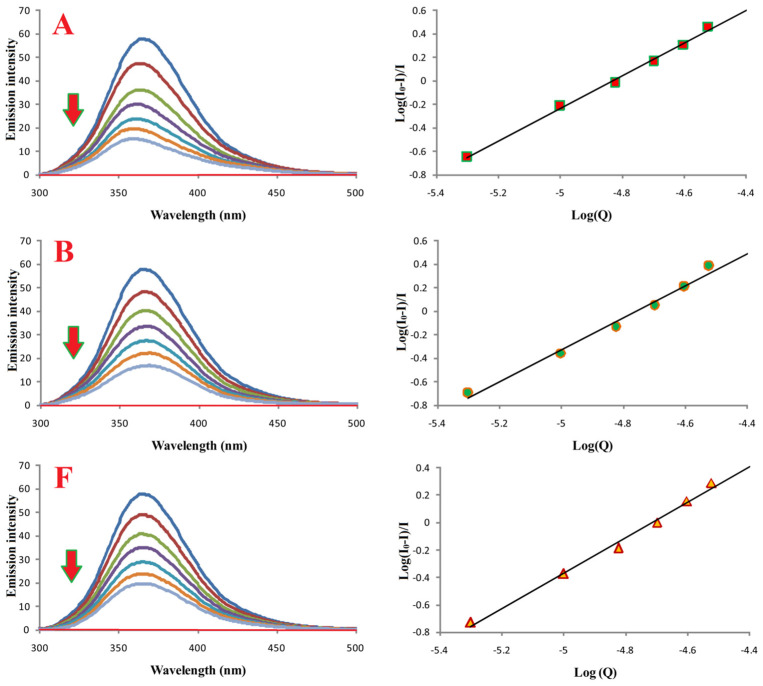
Binding properties of compounds **A**, **B**, and **F** with BSA. Left: emission spectra of BSA in the absence (most intense blue lines) and presence of compounds **A**, **B**, and **F**. Red lines correspond to buffer with the respective compound. [BSA] = 10.0 μM; [**A**, **B**, or **F**] = 0.0–30.0 μM; pH = 7.4; λ_ex_ = 280 nm.

Fluorimetric titration was used to investigate binding of compounds **A**, **B**, and **F** to bovine serum albumin (BSA). Emission spectra (300–500 nm; λ_ex_ 280 nm) are shown in Figure 8. Increasing ligand concentrations resulted in progressive quenching of BSA fluorescence.

Binding constants (K_a_) and number of binding sites (n), calculated from log[(I_0_ − I)/I] versus log[Q] plots, are summarized in Table 3. All compounds formed stable complexes with BSA, and n ≈ 1 for all, indicating a 1:1 binding stoichiometry.

### 3.8. Molecular Docking of Compounds **A**, **B**, and **F** to Bovine Serum Albumin

Molecular docking simulations were performed using the BSA crystal structure co-crystallized with naproxen (NPX) [34]. Binding sites were defined around TRP134 and TRP213, the two tryptophan residues responsible for BSA intrinsic fluorescence. NPX was redocked for validation (RMSD: 1.08 Å).

**Figure 9:**
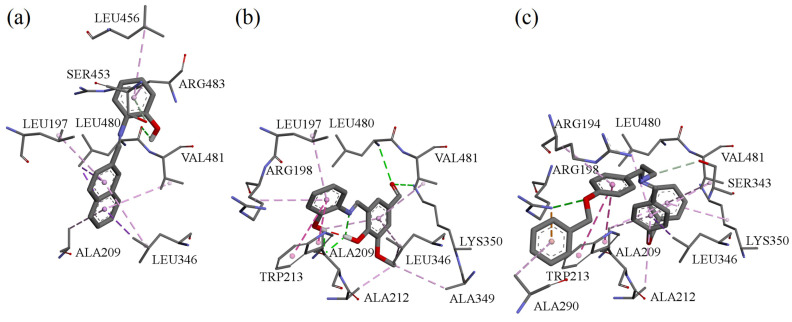
Interactions in the vicinity of TRP213 residue of the BSA molecule, derived from docking experiments: (**a**) compound **A**; (**b**) compound **B**; (**c**) compound **F**.

Estimated binding energies for all compounds at both sites are listed in Table 4. At TRP134, none of the tested molecules interacted directly with the tryptophan residue, whereas at TRP213, all tested ligands docked in close proximity to it. Interactions with BSA in the vicinity of TRP213 for these compounds are shown in Figure 9.

## 4. Discussion

The cytotoxicity results indicate that the synthesized Schiff bases exhibit mild to moderate antiproliferative effects against the studied cancer cell lines, with HeLa cells being the most responsive. The selectivity observed—lower toxicity toward MRC-5 cells—suggests that these compounds may act preferentially on malignant cells.

The structure–activity relationship (SAR) of the synthesized Schiff bases (**A**–**H**) was analyzed based on their cytotoxic activity against HeLa, LS174T, and A549 cancer cell lines, as well as normal MRC5 fibroblasts. Among the mono-imine Schiff bases, compounds **A** and **B** exhibited the most pronounced cytotoxic activity, particularly against HeLa cells, while showing markedly reduced toxicity toward MRC5 cells. The enhanced activity of compound **A** can be attributed to the presence of a naphthyl moiety, which increases molecular planarity and π-conjugation, potentially facilitating interactions with intracellular targets such as DNA. In the case of compound **B**, the combination of phenolic –OH and methoxy substituents may improve hydrogen-bonding capability and modulate electronic distribution within the imine framework, leading to increased antiproliferative activity. In contrast, compound **C**, bearing two phenolic hydroxyl groups on a simple phenyl ring, was completely inactive toward all tested cell lines, suggesting that excessive polarity and reduced lipophilicity may impair cellular uptake. Similarly, compound **D**, despite containing methoxy and hydroxy substituents, displayed only weak activity, indicating that subtle changes in substitution pattern and molecular geometry significantly influence biological response.

The bis-imine Schiff bases (**E**–**H**) showed variable cytotoxic profiles. Compound **F**, containing bulky benzyloxy substituents, exhibited most pronounced activity, which may be associated with increased lipophilicity and enhanced membrane permeability. Compound **E**, featuring iodine substituents, showed limited selectivity and moderate cytotoxicity, possibly due to increased molecular weight and steric effects. Notably, compound **G**, bearing strong electron-donating dimethylamino groups, was completely inactive, indicating that excessive electron donation and reduced planarity may adversely affect interaction with biological targets. Compound **H**, containing hydroxy and methoxy substituents, displayed moderate cytotoxicity but reduced selectivity toward normal cells.

Overall, the SAR analysis suggests that molecular planarity, balanced lipophilicity, and the presence of appropriately positioned hydrogen-bonding groups play key roles in determining cytotoxic potency and selectivity.

Cell cycle analyses revealed that all three selected compounds (**A**, **B**, **F**) induced G1-phase arrest and increased the sub-G1 population, indicating potential pro-apoptotic effects. Compound **B** demonstrated particularly strong influence on cell cycle progression, including an increase in S-phase cells, suggesting interference with DNA replication or checkpoint regulation. Compound **F** additionally caused G2/M arrest after 24 h, which may reflect engagement with mitotic regulatory pathways. These results support the rationale for targeting cell cycle regulation as an anticancer strategy [35,36,37].

All three tested compounds decreased intracellular ROS levels in HeLa cells in a dose-dependent manner. Since elevated ROS in cancer cells contributes to genomic instability, metabolic alterations, and proliferation advantages [38], the reduction of ROS may compromise the oxidative stress–mediated survival mechanisms characteristic of tumor cells. Whether this effect directly contributes to the cytotoxicity observed will require further mechanistic studies.

The comet assay confirmed that even subtoxic concentrations (IC_20_) of compounds **A**, **B**, and **F** were sufficient to induce DNA damage. This suggests that DNA integrity is a potential intracellular target for these Schiff bases, consistent with reports that imine derivatives can interact with or disrupt DNA structure [39].

Western blot analysis further revealed compound-induced downregulation of HIF-1α and PDK3. Given the central role of HIF-1 in metabolic reprogramming under hypoxic and pseudohypoxic conditions [40,41], and the importance of PDK3 in inhibiting pyruvate entry into the TCA cycle [42,43,44], decreased expression of these proteins suggests that the tested compounds may impair cancer cell adaptation to low-oxygen environments and alter metabolic homeostasis. It should be noted that β-actin levels were substantially reduced in cells treated with compound **B**, likely due to high cytotoxicity. As a result, interpretation of normalized HIF-1α and PDK3 levels for compound **B** should be made with caution. Future studies using alternative loading controls (e.g., GAPDH or total protein normalization) will be conducted to confirm these findings. The differential responses among compounds **A**, **B**, and **F** also highlight the importance of structural features governing interactions with HIF-1/PDK pathways.

Binding studies and molecular docking consistently indicated that all compounds interact with BSA with high affinity (K_a_ in the 10^6^ M^−1^ range) and a 1:1 stoichiometry. Protein binding is a key parameter in early pharmacokinetic evaluation, as it influences drug distribution, bioavailability, and half-life [45,46,47,48]. The docking simulations corroborated the experimental data and offered visualization of possible binding modes. The compounds showed moderate selectivity between the two tryptophan-adjacent binding regions, in contrast with naproxen, which favors the TRP213 site. The smaller energy differences between the two sites suggest that the tested Schiff bases bind with lower site-selectivity than NPX.

Overall, the combined biological, biochemical, and computational results indicate that Schiff bases **A**, **B**, and **F** possess multi-target anticancer potential, affecting oxidative stress, DNA integrity, cell-cycle progression, and hypoxia-driven metabolic pathways. Further studies, including mechanistic assays and in vivo evaluation, are warranted to fully elucidate their therapeutic relevance.

## 5. Conclusions

In this study, a series of structurally diverse Schiff bases was evaluated to assess their anticancer properties and underlying mechanisms of action. Compounds **A**, **B**, and **F** demonstrated the strongest antiproliferative effects, with preferential toxicity toward malignant cells. Mechanistic analyses revealed that these derivatives disrupt cell-cycle progression, induce DNA damage, and lower intracellular ROS levels, collectively suggesting engagement of apoptosis-related pathways. All three compounds also decreased the expression of HIF-1α and PDK3, indicating interference with hypoxia-driven metabolic adaptation, a process frequently exploited by tumor cells. Binding studies and docking simulations confirmed high-affinity interactions with serum albumin, supporting their potential for favorable transport and pharmacokinetic behavior. Overall, the results highlight the multi-target biological profile of these Schiff bases and identify them as promising scaffolds for further optimization and development of novel anticancer agents. Future studies should investigate their molecular targets in greater detail and evaluate their in vivo activity and safety.

## Figures and Tables

**Table 1:** Cytotoxic activity (IC_50_ values) of Schiff bases and cisplatin against HeLa, MRC5, LS174T, and A549 cells. (72 h exposure).

Compounds	HeLa IC_50_ (μM)	MRC5 IC_50_ (μM)	LS 174T IC_50_ (μM)	A549 IC_50_ (μM)
**A**	39.45 ± 0.2	>200	82.35 ± 16.81	79.1 ± 4.76
**B**	30.78 ± 2.67	114.07 ± 19.03	107.32 ± 2.23	116.03 ± 29.65
**C**	>200	>200	>200	>200
**D**	102.29 ± 1.54	>200	>200	>200
**E**	78.22 ± 0.21	73.25 ± 6.69	146.49 ± 15.61	>200
**F**	55.73 ± 1.4	113.84 ± 15.13	134.08 ± 39.17	116.85 ± 37.26
**G**	>200	>200	>200	>200
**H**	130.68 ± 6.79	69.0 ± 2.67	118.89 ± 2.18	163.23 ± 10.6
**cisPt**	4.91 ± 0.74	9.35 ± 1.29	5.54 ± 1.03	13.21 ± 0.89

IC_50_ values (μM) were expressed as the mean ± SD determined from the results of MTT assay in three independent experiments.

**Table 2:** Cytotoxic activity (IC_50_ values) of tested compounds against HeLa cells (24 h exposure).

Compounds	HeLa IC_50_ (μM)
**A**	36.12 ± 0.73
**B**	26.79 ± 1.5
**F**	15.41 ± 1.23

IC_50_ values (μM) were expressed as the mean ± SD determined from the results of MTT assay in three independent experiments.

**Table 3:** Binding parameters (Ka, n) and correlation coefficients for interactions of compounds **A**, **B**, and **F** with BSA.

Compound	K_a_ [M^−1^]	n	R
**A**	(5.0 ± 0.2) × 10^6^	1.3	0.997
**B**	(2.9 ± 0.2) × 10^6^	1.4	0.990
**F**	(1.2 ± 0.2) × 10^6^	1.2	0.996

**Table 4:** Estimated free energies of binding (E), derived from docking simulations.

Compound	E [kcal mol^−1^]	
	TRP134	TRP213
**A**	−8.38	−7.97
**B**	−7.22	−8.13
**F**	−9.42	−8.97
NPX	−6.54	−8.60

## Data Availability

The original contributions presented in this study are included in the article/Supplementary Material. Further inquiries can be directed to the corresponding author.

## Supplementary Materials

The following supporting information can be downloaded at https://www.mdpi.com/article/10.3390/cimb48010057/s1.
