# Supplementary material for: Multi-Target Anticancer Activity of Structurally Diverse Schiff Bases: Insights into Cell-Cycle Arrest, DNA Damage, Metabolic Signaling, and Biomolecular Binding
Source: Curr Issues Mol Biol. 2026 Jan 1;48(1):57. doi: 10.3390/cimb48010057 (PMC12840311; doi:10.3390/cimb48010057)
Supplement: Supplementary file 1 [file cimb-48-00057-s001.zip › cimb-4058640-supplementary.pdf]

Supplementary Material associated with the paper

Multi-Target Anticancer Activity of Structurally Diverse Schiff Bases: Insights into Cell-Cycle Arrest, DNA Damage, Metabolic Signaling, and Biomolecular Binding

Nenad Joksimović,[1] Jelena Petronijević,[1] Ignjat Filipović,[1] Nenad Janković,[2] Bojana Ilić,[3] Tatjana Stanojković,[4] and Ana Djurić[3]*

[1] University of Kragujevac, Faculty of Science, Department of Chemistry, Radoja Domanovića 12, 34000 Kragujevac, Serbia.

[2] University of Kragujevac, Institute for Information Technologies Kragujevac, Department of Sciences, Jovana Cvijića bb, 34000 Kragujevac, Serbia.

[3] Clinic for Endocrinology, Diabetes and Metabolic Diseases, University Clinical Centre of Serbia, Belgrade, Serbia.

[4] Institute of Oncology and Radiology of Serbia, Pasterova 14, 11000 Belgrade, Serbia.

Corresponding author's e-mail address: nenad.joksimovic@pmf.kg.ac.rs;

Corresponding author's postal address: Faculty of Science, University of Kragujevac, Department of Chemistry, P.O. Box 60, 34000 Kragujevac, Serbia

Corresponding author's telephone and fax numbers: +381 34 336362

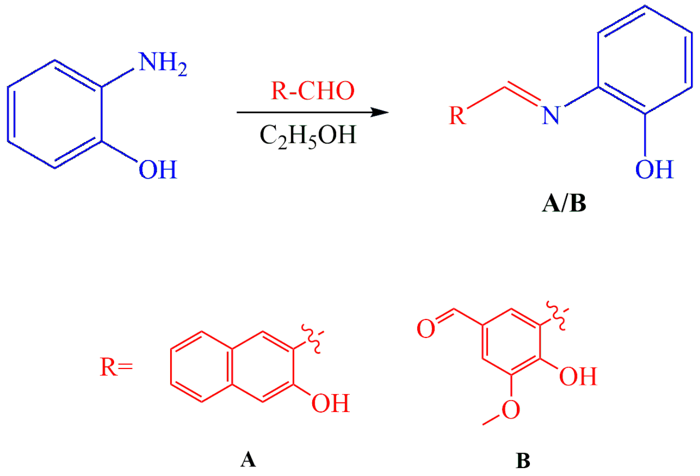

Scheme S1. Synthetic procedures for the synthesis of novel Schiff bases **A** and **B**.

(*E*)-3-(((2-hydroxyphenyl)imino)methyl)naphthalen-2-ol (**A**)

Orange powder; yield: 94%; mp = 252 °C; FT-IR (KBr, ν, cm^{-1}): 3429 (br, O–H), 3026 (Ar–H), 2424 (H-bonded O–H), 2175 (Ar overtone), 1631 (C=N), 1548, 1460 (Ar C=C), 1356, 1240 (C–O), 1141 (C–N); ^{1}H NMR (200 MHz, DMSO-d_6) δ 15.73 (d, 1H, OH), 10.39 (s, 1H, OH), 9.50 (d, 1H, CH=N), 8.39 (d, J = 8.5 Hz, 1H, CH_{Ar}), 7.94 (d, J = 7.0 Hz, 1H, CH_{Ar}), 7.79 (d, J = 9.4 Hz, 1H, CH_{Ar}), 7.66 (d, J = 7.7 Hz, 1H, CH_{Ar}), 7.47 (t, J = 7.0 Hz, 1H, CH_{Ar}), 7.25 (t, J = 7.3 Hz, 1H, CH_{Ar}), 7.16–6.90 (m, 3H, CH_{Ar}), 6.79 (d, J = 9.4 Hz, 1H, CH_{Ar}) ppm. ^{13}C NMR (50 MHz, DMSO-d_6) δ 177.67, 149.67, 148.63, 138.07, 134.08, 129.15, 128.83, 128.26, 126.92, 126.03, 125.20, 123.20, 120.00, 119.86, 117.78, 116.15, 107.90 ppm. Calcd. for $C_{17}H_{13}NO_2$ (%): C 77.55, H 4.98, N 5.32; found: C 77.59, H 4.97, N 5.34.

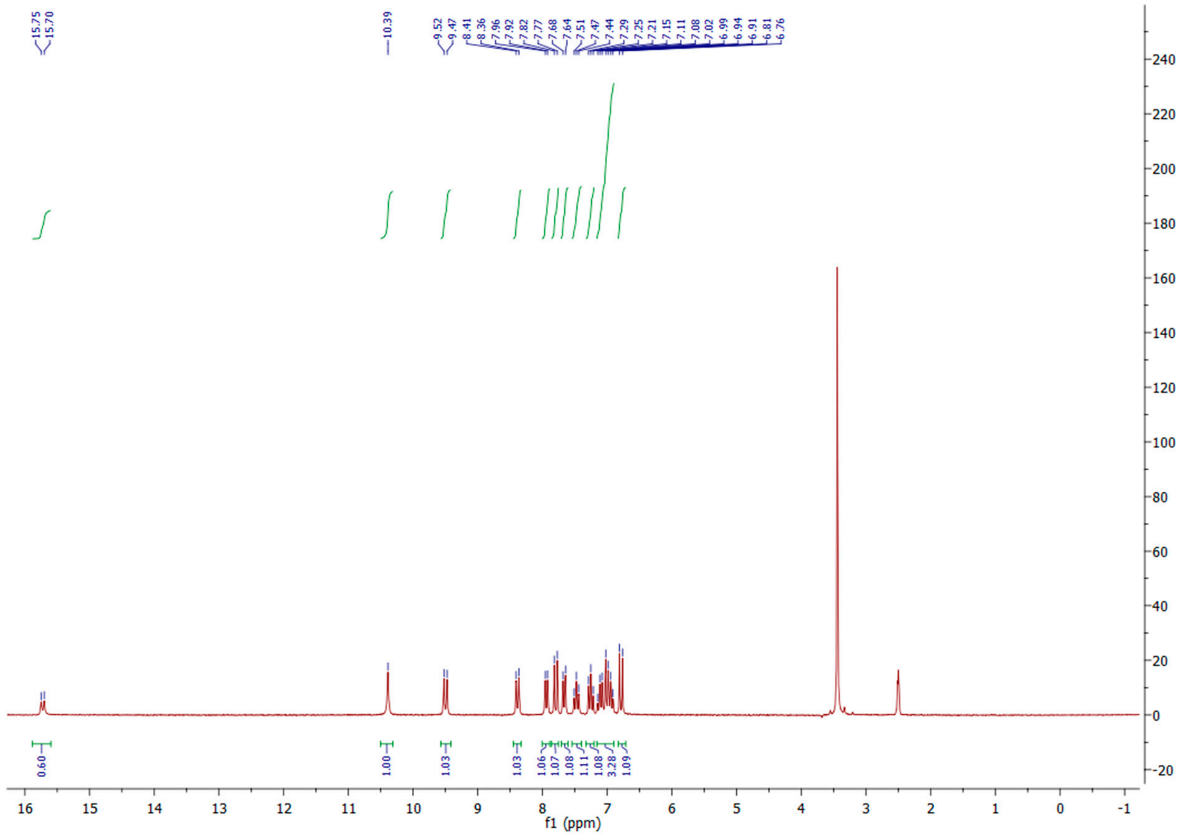

Fig. S1 ^{1}H NMR spectrum of compound **A**

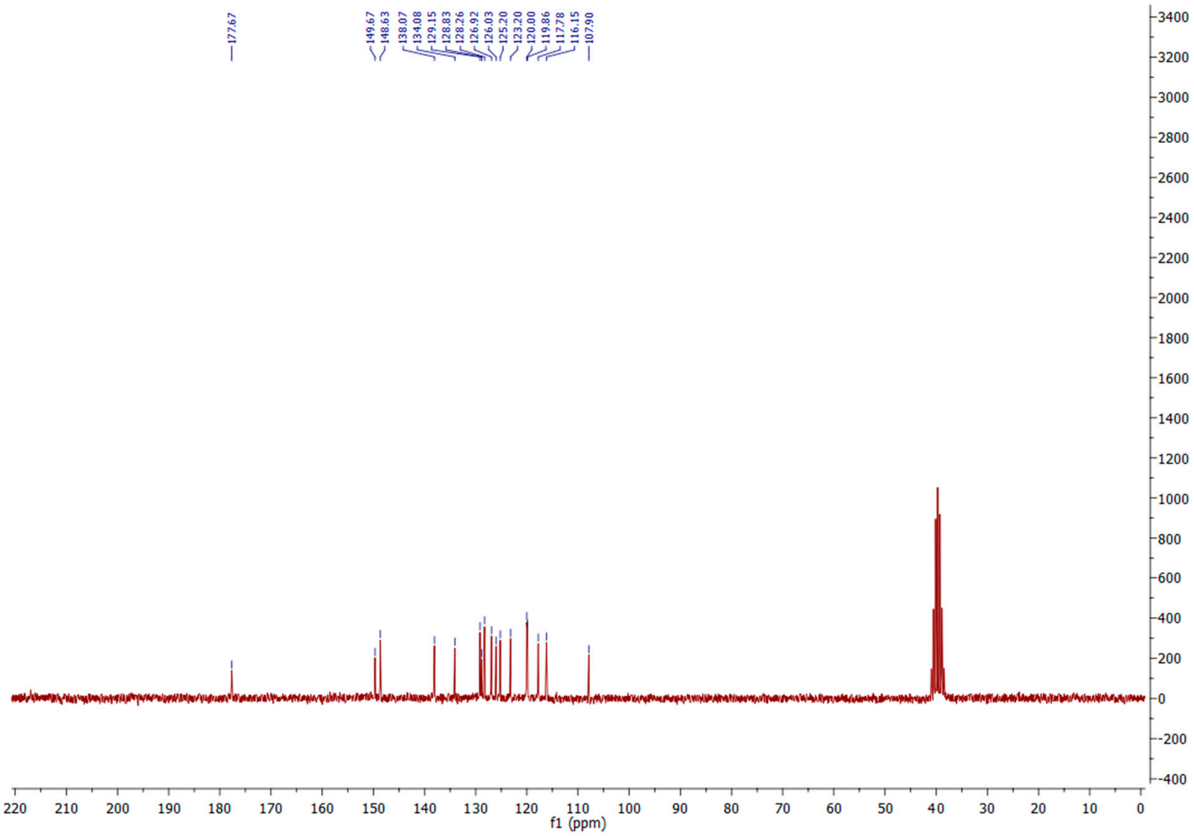

Fig. S2 ^{13}C NMR spectrum of compound **A**

3-((E)-(2-hydroxyphenylimino)methyl)-4-hydroxy-5-methoxybenzaldehyde (**B**)
Orange powder; yield: 89%; mp = 163 °C; FT-IR (KBr, v, cm^{-1}): 3428 (br, O–H), 2926 (C–H), 1673 (C=O), 1617 (C=N), 1477 (Ar C=C), 1362, 1284 (C–O), 1150 (C–O–C); ^{1}H NMR (200 MHz, DMSO-d_6) δ 15.53 (s, 1H, OH), 10.55 (s, 1H, OH), 9.67 (s, 1H, CHO), 9.20 (s, 1H, CH=N), 7.82–7.53 (m, 2H, CH_{Ar}), 7.27-7.13 (m, 2H, CH_{Ar}), 6.98 (m, 2H, CH_{Ar}), 3.80 (s, 3H, OCH_3) ppm; ^{13}C NMR (50 MHz, DMSO-d_6) δ 189.71, 169.14, 158.29, 151.80, 149.61, 134.04, 128.81, 128.22, 124.42, 120.08, 118.34, 116.59, 115.39, 108.73, 55.59 ppm. Calcd for $C_{15}H_{13}NO_4$ (%): C 66.41, H 4.83, N 5.16; found: C 66.49, H 4.82, N 5.17.

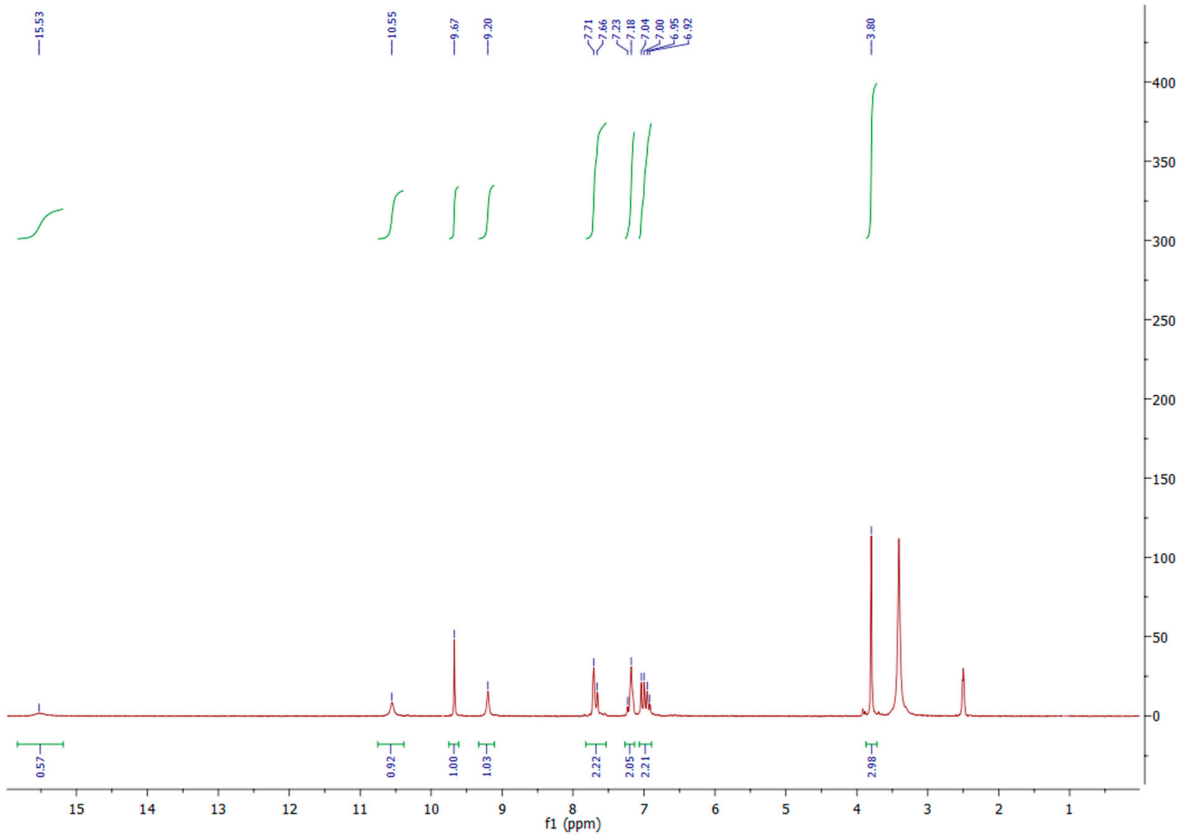

Fig. S3 ^{1}H NMR spectrum of compound **B**

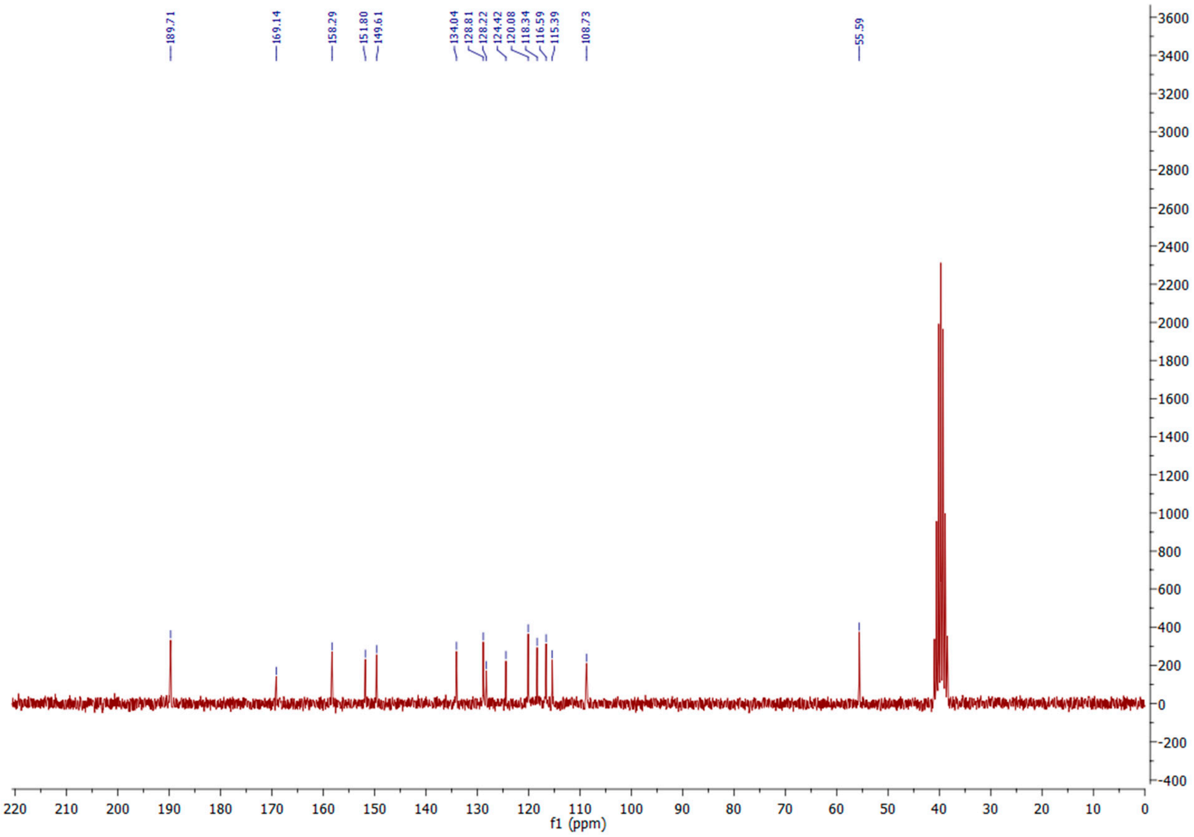

Fig. S4^{13}C NMR spectrum of compound **B**

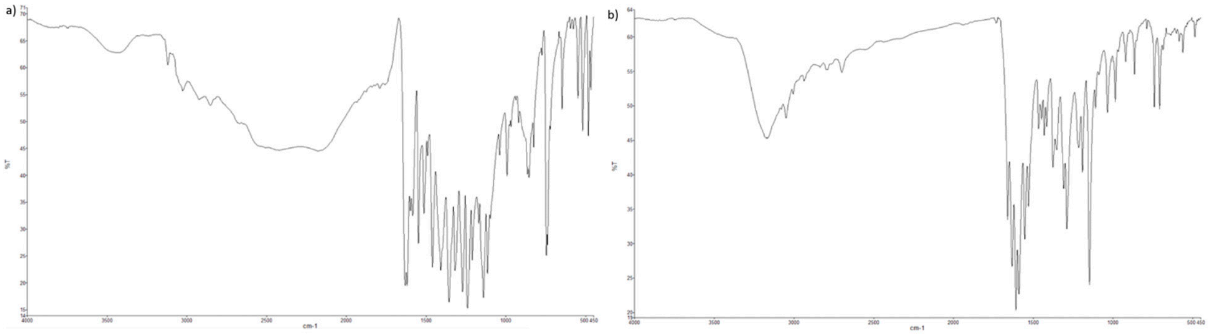

Fig S5. FTIR spectra of compounds **A** (a) and **B** (b).
